# Evaluation of Glycemic Control in Patients With Diabetes by a Continuous Glucose Monitoring System During the Month of Ramadan

**DOI:** 10.7759/cureus.72710

**Published:** 2024-10-30

**Authors:** Nassim Essabah Haraj, Nana Aichatou Harouna Malam Brah, Siham Elaziz, Asma Chadli

**Affiliations:** 1 Department of Endocrinology and Metabolic Diseases, Ibn Rochd University Hospital, Casablanca, MAR; 2 Laboratory of Neurosciences and Mental Health, Faculty of Medicine and Pharmacy, University Hassan II, Casablanca, MAR

**Keywords:** continuous glucose monitoring systems, diabetes, fasting, glycemic control, ramadan

## Abstract

Introduction: Ramadan presents challenges for patients with type 2 diabetes mellitus (T2DM) who choose to fast. Maintaining good glycemic control through regular monitoring during fasting is crucial. This study aims to evaluate and compare glycemic fluctuations in fasting and non-fasting T2DM patients during Ramadan.

Materials and methods: We conducted a prospective comparative study on 39 T2DM patients, divided into fasting and non-fasting groups based on the International Diabetes Federation - Diabetes and Ramadan Alliance (IDF-DAR) Practical Guidelines 2021. Each patient wore an iPro®2 Continuous Glucose Monitoring System (CGMS) device (Medtronic plc, Dublin, Ireland), for five to seven days, with consultations scheduled before, during, and after Ramadan. Glycated hemoglobin (HbA1c) levels were measured before and after Ramadan. The fasting risk score was calculated using the DAR guidelines.

Results: The fasting group included 29 fasting patients with an average fasting risk score of 2.7 according to the DAR guidelines, while the non-fasting group had 10 non-fasting patients with an average score of 6.55. The average HbA1c was estimated by the CGMS at 7.36% for fasting patients and 7.1% for non-fasting patients. In the fasting group, 80% of patients experienced at least one episode of hyperglycemia, averaging 4.03 episodes per day, predominantly occurring during pre-dawn (suhoor) and post-sunset (iftar) periods. Hypoglycemia occurred in 24% of fasting patients, mainly before iftar. In contrast, the non-fasting group had a higher average of 6.8 hyperglycemic episodes per day, with 50% of the group also experiencing hypoglycemia. Notably, a significant improvement in HbA1c was observed in the non-fasting group after Ramadan (7.77 ± 0.89 vs. 9.13 ± 1.99, p=0.037). No significant changes in HbA1c, weight, or body mass index were found in the fasting group.

Conclusion: Pre-Ramadan education, risk stratification, and continuous monitoring during Ramadan are essential to prevent metabolic complications in T2DM patients, whether fasting or non-fasting. CGMSs reveal a high prevalence of hyperglycemia, especially in non-fasting patients, underscoring the need for tailored treatment adjustments to achieve optimal glycemic control.

## Introduction

Type 2 diabetes mellitus (T2DM) is a complex metabolic disorder marked by both insulin resistance and impaired insulin production. Its global prevalence is steadily increasing, largely due to rising obesity rates, sedentary lifestyles, and poor dietary habits. Effective management of T2DM hinges on maintaining optimal glycemic control to prevent complications affecting both small and large blood vessels. Continuous glucose monitoring systems (CGMS) have transformed diabetes management by offering real-time, detailed glucose readings, which allow for more precise and proactive treatment adjustments. This technology is especially valuable during Ramadan, a month when Muslims fast from sunrise to sunset, presenting unique challenges in maintaining glycemic stability [1].

Fasting has no demonstrated adverse consequences when the faster is in good health while fasting diabetic patients are exposed to the risks of hypoglycemia, hyperglycemia, and dehydration during the fast [2,3].

Recent studies have looked at some of the changes that can occur as a result of fasting during Ramadan: weight changes, blood sugar imbalance, and metabolic disturbances [4,5]. The patient with diabetes who wishes to observe the fast risks unbalancing their diabetes control. In the absence of monitoring and therapeutic adaptation before the month of Ramadan, fasting in patients with diabetes can be dangerous and associated with an increase in complications and a glycemic imbalance [6]. Continuous measurement of blood glucose to assess this imbalance is a promising technique. Indeed, it is a necessary technology in patients with diabetes to study unstable diabetes, detect asymptomatic and nocturnal hypoglycemia, and thus allow treatment to be adapted [7].

The International Diabetes Federation - Diabetes and Ramadan Alliance (IDF-DAR) Practical Guidelines 2021 developed a new fasting risk score in patients with diabetes and management recommendations for this month for fasting patients in order to support patients for a risk-free fast [8]. The objective of our study is to evaluate the impact of fasting on glycemic balance with a CGMS in fasting and non-fasting patients with type 2 diabetes, after having benefited from structured education and an adaptation of their treatment before Ramadan. We hypothesize that structured education and treatment adaptation before Ramadan will enable fasting patients with diabetes to maintain glycemic control similar to that of non-fasting patients, minimizing the risk of complications.

The primary endpoint of our study is the change in glycated hemoglobin (HbA1c) levels from baseline to the end of Ramadan. Secondary endpoints include the frequency of hypoglycemic and hyperglycemic episodes, time in range, weight changes, and the incidence of acute complications.

## Materials and methods

This prospective longitudinal study was conducted at the Department of Endocrinology and Metabolic Diseases, Ibn Rochd University Hospital, Casablanca, during Ramadan 2021 (April and May 2021). The study was approved by the Ethics Committee of the Centre Hospitalier Universitaire (CHU) Ibn Rochd (Reference Number: 424). Free and informed consent of patients was obtained before inclusion in the study. All information collected was kept confidential and anonymous at all stages, with respect to the Declaration of Helsinki. The study data was securely stored, with no identifying information collected.

Sample selection

The patients included in the study were classified according to the IDF-DAR recommendations which categorize individuals into low, moderate, and high-risk groups depending on their diabetes status, risk of complications, and previous fasting experience. Patients were divided into three groups based on their IDF-DAR risk score: low-risk fasting (patients with a score of 0-3), moderate-risk fasting (patients with a score of 3.5-6), and high-risk fasting (patients with a score above 6) [8]. Subsequently, they were grouped into two larger categories: Group 1 (fasting group) which comprised adult patients with T2DM who were authorized to fast (low risk and moderate risk) and consented to participate in the study, and Group 2 (non-fasting group) which included patients with T2DM with a contraindication to fasting (high-risk group).

We excluded non-consenting patients, patients with type 1 diabetes, pregnant women, and patients under 18 years of age from the study for ethical reasons and due to the vulnerability of these populations.

Data collection

The data collected include demographic characteristics (age, sex, marital status, social coverage), the characteristics of diabetes (the duration of diabetes, the treatment regimen, degenerative complications, body mass index (BMI), weight, height, HBA1c, glycemic self-monitoring), parameters related to fasting in the month of Ramadan (food survey during the month of Ramadan, IDF-DAR fasting risk score), and the interpretation parameters of the CGMS (time in target, time above target, time below target, glycemic excursions, HBA1c estimated by the CGMS).

Intervention

During the first consultation, patients were seen before the month of Ramadan. They underwent an evaluation of glycemic balance by HbA1c and self-monitoring of capillary glucose. Patients were classified as fasting or non-fasting, with appropriate adaptation of their treatment and education on precautions to be taken when fasting during Ramadan. Nutritional recommendations for diabetes management during Ramadan included a late suhoor with complex carbohydrates and fiber-rich foods, a moderate iftar with a balanced meal, and limiting high sugar and high-fat foods to maintain stable blood glucose levels. Adequate hydration was emphasized, especially during non-fasting hours. During the subsequent second consultation, the iPro® 2 Professional CGMS device (Medtronic plc, Dublin, Ireland) was installed, which patients wore for five to seven days; this CGMS measures interstitial glucose levels, which were cross-compared with self-monitored capillary glucose. Throughout this period, patients maintained a weekly diary to assess their eating behavior. During the third consultation, patients received an analysis of the continuous blood glucose recordings. Based on these results, adjustments were made to their treatments, and a reassessment of the risks associated with fasting. Finally, during the fourth consultation after the month of Ramadan, patients underwent another evaluation of glycemic balance by HbA1c and self-monitoring of capillary glucose.

Statistical analysis

Statistical analysis was performed using the IBM SPSS Statistics for Windows, Version 25.0 (Released 2017; IBM Corp., Armonk, New York, United States). Descriptive statistics were calculated for continuous and categorical variables. Comparisons between groups were made using the Chi-square test for categorical data and the Student’s t-test or Mann-Whitney U test for continuous data, depending on normality assumptions. A p-value < 0.05 was considered statistically significant.

## Results

The study included 39 patients of which 29 were fasting and 10 were non-fasting patients. Table 1 combines the demographic and diabetes-related characteristics of the two groups of patients. In terms of antidiabetic treatment, 23 patients were on dual therapy: 20 on metformin-sulfonamides and three on metformin-dipeptidyl peptidase 4 (DPP4) inhibitors.

**Table 1:** Characteristics of fasting and non-fasting diabetic patients ODT: oral antidiabetic treatment

	Fasting Group (n=29)	Non-Fasting Group (n=10)	p-value
Sex, n (%)			<0.001
Female	21 (72.4%)	6 (60%)	
Male	8 (27.6%)	4 (40%)	
Average Age (years), mean±SD (range)	55.03 ± 8.902 (37 – 71)	61.6 ± 7.64 (48 – 72)	0.053
Marital Status, n (%)			<0.001
Single	2 (6.9 %)	0 (0%)	
Married	21 (72.4%)	7 (70%)	
Divorced	3 (10.35%)	1 (10%)	
Widower / Widow	3 (10.35%)	2 (20%)	
Mutual Insurance Coverage, n (%)			<0.001
Yes	24 (82.7 %)	10 (100%)	
No	5 (17.3 %)	0 (0%)	
Environment, n (%)			0.744
Urban	28 (26.5 %)	10 (100%)	
Rural	1 (3.5 %)	0 (0%)	
Antecedents, n (%)			0.005
Yes	15 (51.7%)	10 (100%)	
No	14 (48.3%)	0 (0%)	
Dyslipidemia, n (%)			0.323
Yes	7 (24.1%)	1 (10%)	
No	22 (75.9%)	9 (90%)	
Arterial hypertension, n (%)			0.620
Yes	17 (58.6%)	6 (60%)	
No	12 (41.4%)	4 (40%)	
Previous surgery, n (%)			0.419
Yes	6 (20.7%)	3 (30%)	
No	23 (79.3%)	7 (70%)	
Average duration of diabetes (years)	7.32 ± 3.96 (0-18)	10.4 ± 5.48 (4-18)	0.184
Diabetic treatment, n (%)			0.044
Diet and Hygiene Rules	1 (3.5%)	1 (10%)	
Monotherapy	7 (24%)	3 (30%)	
Bi-therapy	19 (65.5%)	4 (40%)	
Mixed (ODT + Insulin)	2 (7%)	0 (0%)	
Insulin, n (%)			<0.001
Yes	2 (7%)	5 (50%)	
No	27 (93%)	5 (50%)	
Diabetic retinopathy, n (%)			<0.001
Yes	3 (10.35%)	10 (100%)	
No	26 (89.65%)	0 (0%)	
Diabetic nephropathy, n (%)			0.6
Yes	2 (7%)	1 (10%)	
No	27 (93%)	9 (90%)	
Diabetic neuropathy, n (%)			0.001
Yes	12 (41.4%)	10 (100%)	
No	17 (58.6%)	0 (0%)	
Heart failure, n (%)			0.001
Yes	2 (7%)	6 (60%)	
No	27 (93%)	4 (40%)	
Cerebrovascular accident, n (%)			<0.001
Yes	1 (3.5%)	6 (60%)	
No	28 (96.5%)	4 (40%)	
Obliterative arteriopathy of the lower limbs, n (%)			0.13
Yes	0 (0%)	3 (30%)	
No	29 (100%)	7 (70%)	

Table 2 summarizes the characteristics related to glycemic control in fasting and non-fasting patients, and reveals that non-fasting patients had significantly higher HbA1c levels before Ramadan compared to fasting patients. After Ramadan, HbA1c levels improved in non-fasting patients. The average number of calories consumed during fasting days was lower in fasting patients.

**Table 2:** Characteristics related to glycemic control Hba1C: glycated hemoglobin, IDF-DAR: International Diabetes Federation-Diabetes and Ramadan Alliance Practical Guidelines 2021 ; BMI: body mass index

	Fasting Group (n=29 patients), mean±SD (range)	Non-Fasting Group (n=10), mean±SD (range)	P-value
HbA1c before Ramadan	7.34 ± 0.9 (5.9 – 9)	9.13 ± 1.99 (6.7 – 12.6)	0.009
HbA1c after Ramadan	7.51 ± 0.97 (6 – 9.5)	7.77 ± 0.89 (6 – 9)	0.411
IDF-DAR fasting risk score	2.1 ± 4.98 (0.5 – 4.5)	6.55 ± 1.73 (4 – 9.5)	<0.001
Average number of calories consumed during a fasting day	1167 ± 251.58 (710 – 1715 )	1330 ± 212.39 (1060 – 1800)	0.03
Weight before Ramadan	74.94 ± 13.64 (47 – 99.4)	77.33 ± 11.37 (58 – 96)	0.456
BMI before Ramadan	28.03 ± 4.98 ( 21 – 39.8)	28.69 – 3.11 (24.96 – 33.21)	0.326
Weight after Ramadan	75.77 ± 13.03 (46 – 100)	77.07 ± 12.04 (57 – 98)	0.676
BMI after Ramadan	29.11 ± 5.08 (21 – 38.6)	28.52 ± 3.4 (24 – 33.9)	0.347

Table 3 summarizes the results of the interpretation of the CGMS. In the fasting group, 80% of patients experienced at least one episode of hyperglycemia, with an average of 4.03 episodes per day, primarily occurring during the pre-dawn (suhoor) and post-sunset meals (iftar). In contrast, the non-fasting group had a higher average of 6.8 hyperglycemic episodes per day, with 50% of the group also experiencing hypoglycemia. We observed that the number of hyperglycemic and hypoglycemic events was significantly higher in non-fasting patients compared to those who were fasting. Hyperglycemic events were particularly frequent around the time of iftar. Conversely, fasting patients had a greater proportion of time spent within the target glucose range.

**Table 3:** Comparison of participant characteristics according to CGMS Hba1C: glycated haemoglobin; CGMS: continuous glucose monitoring system

	Fasting Group (n=29), mean±SD (range)	Non-Fasting Group (n=10), mean±SD (range)	P-value
Numbers of hypoglycemia	0.25 ± 0.518 ( 0 – 2)	1.8 – 2.34 ( 0 – 7)	0.022
Numbers of hyperglycemia	4.03 ± 3.07 (0 – 12)	6.8 – 2.3 (3 – 10)	0.008
Hba1c estimated by CGMS (%)	7.36 ± 1.27 (5.7 – 10)	7.1 ± 0.89 (5.9 – 8.6)	0.711
Time in target range	77.07 ± 24.77 (5-100)	64.2 ± 17.41 (41 – 91)	0.047
Time spent above target	22.69 ± 24.86 (0 – 95)	31.7 ± 16.95 (7 – 59)	0.098
Time spent below target	0.28 ± 0.64 (0 – 3)	4.2 – 8.02 (0 – 26)	0.008
Average blood sugar (mg/dl)	156.3 ± 35.24 (101 – 248)	157 ± 25.44 (123 – 199)	0.711
Coefficient of variation	27.31 ± 12.25 (14 – 78)	31.6 ± 8.22 (22 – 53)	0.147
Mean standard deviation	41.69 ± 18.95 (18 – 88)	49.4 ± 10.83 (35 – 71)	0.119
Interpretation			0.640
Stable diabetes	20 (69%)	7 (70%)	
Unstable diabetes	9 (31%)	3 (30%)	

Table 4 represents the evaluation of the impact of Ramadan on Hba1c and BMI in fasting and non-fasting patients, and shows that while fasting patients did not have significant changes in HbA1c, weight, or BMI after Ramadan, non-fasting patients showed a significant reduction in HbA1c levels post Ramadan.

**Table 4:** Assessment of glycemic control before and after the month of Ramadan in fasting and non-fasting patients Hba1C: glycated hemoglobin; BMI: body mass index

		Before Ramadan, mean±SD (range)	After Ramadan, mean±SD (range)	p-value
Fasting Group (n=29)	Average HbA1c	7.34 ± 0.9 (5.9-9)	7.51 ± 0.97 (6-9.5)	0.274
	Average weight	74.94 ±13.64 (47-99.4)	75.77±13.33 (46-100)	0.455
	Average BMI	28.03 ± 4.98 (21-39.8)	29.11 ± 5.08 (21.3 – 38.6)	0.442
Non-Fasting Group (n=10)	Average HbA1c	9.13 ± 1.99 (6.1 – 12.6)	7.77 ± 0.89 (6-9)	0.037
	Average weight	77.33 ± 11.37 (58 -96)	77.07 ± 12.04 (57 – 98)	0.248
	Average BMI	28.69 ± 3.11 (24.96 – 33.21)	28.52 ± 3.4 (24.06 – 33.9)	0.458

No serious complications were noted in our patients (severe hypoglycemia, diabetic ketosis, stroke, hospitalization). After the interpretation of the CGMS results, the indication to discontinue the fast was made in six patients faced with significant glycemic excursions and the instability of the diabetes.

## Discussion

Fasting during Ramadan presents significant challenges and risks for individuals suffering from diabetes. Despite these difficulties, many individuals with diabetes choose to fast. The main risks include hypoglycemia, hyperglycemia, ketoacidosis, and dehydration [3].

The IDF-DAR 2021 guidelines have made it possible to standardize the management of Ramadan fasting in diabetic patients, through a simple and practical classification. Risk stratification is a crucial aspect of all recommendations concerning diabetes and Ramadan [8,9].

Therefore, it is essential for patients with diabetes to consult their healthcare provider to assess these risks and classify them accordingly. The recommendations of the IDF-DAR include a risk calculator with various variables, allowing all physicians to categorize patients wishing to fast by type and duration of diabetes, presence of hypoglycemia, characteristics of glycemic control, self-monitoring blood glucose, acute complications, chronic complications/comorbidities, pregnancy, frailty and cognitive function, physical activity, previous Ramadan experience, fasting hours, and diabetes treatment [10-14].

According to the Epidemiology of Diabetes and Ramadan (EPIDIAR) study, only 62% of T2DM patients and 68% of T1DM patients receive specialized advice and therapeutic adjustments during the month of Ramadan [3]. A pre-Ramadan consultation is essential for the management of diabetic patients and it allows risk stratification, therapeutic education, and treatment adaptation before this holy month [4,6]. According to the IDF-DAR Practical Guidelines 2021, patients at low or moderate risk can fast after consulting a healthcare professional one to two months before Ramadan and receiving appropriate therapeutic education, with adjustment of their treatment and reinforced follow-up (including self-monitoring of blood glucose). However, the IDF specifies that patients at high risk "should not fast" and that patients at very high risk "must not fast" [15,16]. These recommendations emphasize the importance of a comprehensive medical evaluation before Ramadan for each patient with diabetes, taking into account their individual needs. In cases of clear contraindications, it is the responsibility of the physician to strongly advise against fasting and ensure that the diabetic patient understands and accepts this recommendation.

In the present study, fasting and non-fasting patients benefited from preparation before the month of Ramadan. Studies have shown that in the absence of good preparation, glycemic imbalance and acute complications are more likely to occur [16,17]. The usage of a CGMS allows a good evaluation of glycemic excursions and glycemic variability in patients [17-19]. Shaikh et al. suggest integrating technology such as CGMS and automated insulin delivery systems to manage diabetes more effectively during Ramadan [20].

In our study, fasting patients had a higher time inside the target range compared to non-fasting patients, who had higher numbers of hyperglycemia and a higher time spent below the target range. This observation can be explained by the dietary differences at the time of the iftar and the abstention from eating during the day even in non-fasting patients. Comparatively, the study of Lessan et al., which analyzed the glycemic balance in 53 patients and found a significant change in their mean CGMS curve during Ramadan, with a slow decline in glucose levels during fasting hours and a rapid rise after iftar, overall markers of glucose control (mean interstitial glucose, mean amplitude of glycaemic excursion, high and low blood glucose indices, and hypoglycemia rates) did not show statistically significant changes between fasting and non-fasting periods [21].

No serious complications were noted in our study, and there was no significant difference between HbA1c, weight, and BMI in fasting patients, while improvement in HbA1c was observed in non-fasting patients. This improvement can be explained by the close follow-up (four consultations during the month of Ramadan in our study) and the adaptation of the treatment before the month of Ramadan and after the results of the CGMS.

In six fasting patients in the present study, an indication to discontinue the fast was made when faced with a significant glycemic imbalance in the interpretation of the CGMS data, with the presence of unstable diabetes. Explaining the glycemic imbalance to patients through the CGMS curves helps the patients accept the recommendation to discontinue the fast.

This study's strengths include the rigorous use of CGMS to provide accurate assessments of glycaemic variability and the close follow-up of patients, with four consultations during Ramadan. This close monitoring allowed for timely adjustments to treatment based on CGMS data.

The primary limitation of our study is the small sample size, underscoring the need for larger studies to validate our findings. Additionally, as a single-center study, our results may not be generalizable to other populations. Further studies are recommended to explore the long-term effects of Ramadan fasting on diabetic patients and to refine guidelines for medication adjustments. The integration of technology, such as CGMS and real-time monitoring, can enhance the management of diabetes during Ramadan and other fasting periods [21].

## Conclusions

Pre-Ramadan education, in conjunction with risk stratification and continuous monitoring throughout Ramadan, is integral to preventing metabolic complications in both fasting and non-fasting diabetic patients. Our findings particularly emphasize the higher incidence of hyperglycemia among non-fasting patients, reinforcing the value of CGMS in enabling more precise and individualized treatment adjustments during this period.

Despite the significant insights provided by this study, it is important to recognize its limitations, including the small sample size and the specific demographic of the study population, which may constrain the broader applicability of the findings. Future research should aim to include larger, more heterogeneous populations and assess the long-term effects of these interventions on diabetes management during Ramadan. Addressing these areas will not only enhance our understanding but also refine clinical strategies for optimizing care for patients with diabetes during this critical time.
